# Supplementary figures and images for: eDNA metabarcoding shows highly diverse but distinct shallow, mid-water, and deep-water eukaryotic communities within a marine biodiversity hotspot
Source: PeerJ. 2025 Apr 22;13:e19249. doi: 10.7717/peerj.19249 (PMC12024446; doi:10.7717/peerj.19249)

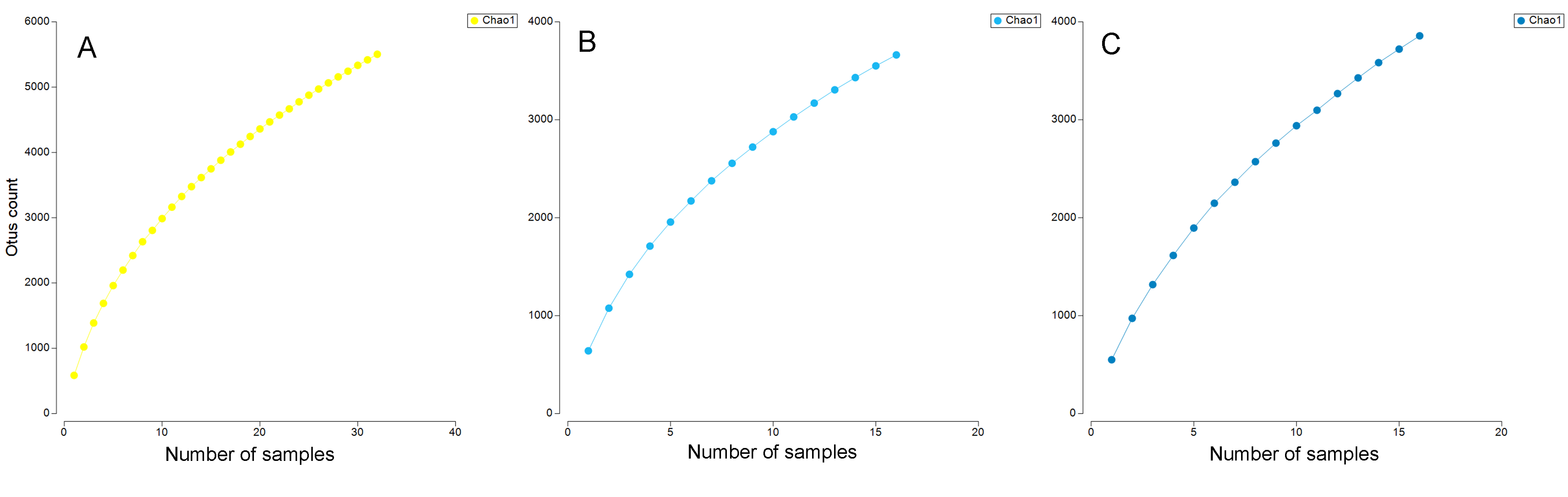

Supplement: Supplemental Information 2 — Chao1 cumulative OTU richness curves per vertical layers. All eDNA sea water samples (A). Shallow samples ¡ 30 m (B). Deep samples, including mid-water 30-150 m and deep-water ¿200 m (C). [file peerj-13-19249-s002.png]
